# Fluid–Structure Interaction Analyses of Biological Systems Using Smoothed-Particle Hydrodynamics

**DOI:** 10.3390/biology10030185

**Published:** 2021-03-02

**Authors:** Milan Toma, Rosalyn Chan-Akeley, Jonathan Arias, Gregory D. Kurgansky, Wenbin Mao

**Affiliations:** 1 Department of Osteopathic Manipulative Medicine, New York Institute of Technology, College of Osteopathic Medicine, Old Westbury, NY 11568, USA; jarias08@nyit.edu (J.A.); gkurgans@nyit.edu (G.D.K.); 2 Lang Research Center, NewYork-Presbyterian Hospital, Queens, NY 11355, USA; roc9166@nyp.org; 3 Department of Mechanical Engineering, University of South Florida, Tampa, FL 33620, USA; wmao@usf.edu

**Keywords:** fluid–structure interaction, biological systems, smoothed-particle hydrodynamics, simulation, numerical analyses

## Abstract

**Simple Summary:**

Computational simulations of biological systems are an intrinsic element of engineering in medicine, allowing physicians the ability to visualize the pathophysiology behind a disease. Many biomedical applications require fluid–structure interaction analyses. This paper provides a review of fluid–structure interaction methods using smoothed-particle hydrodynamics to analyze the dynamics of biological processes.

**Abstract:**

Due to the inherent complexity of biological applications that more often than not include fluids and structures interacting together, the development of computational fluid–structure interaction models is necessary to achieve a quantitative understanding of their structure and function in both health and disease. The functions of biological structures usually include their interactions with the surrounding fluids. Hence, we contend that the use of fluid–structure interaction models in computational studies of biological systems is practical, if not necessary. The ultimate goal is to develop computational models to predict human biological processes. These models are meant to guide us through the multitude of possible diseases affecting our organs and lead to more effective methods for disease diagnosis, risk stratification, and therapy. This review paper summarizes computational models that use smoothed-particle hydrodynamics to simulate the fluid–structure interactions in complex biological systems.

## 1. Introduction

Computational simulations of biomedical applications are an intrinsic element of engineering in medicine, allowing physicians the ability to visualize the pathophysiology behind a disease. A medical professional can use the findings of biomedical simulations to explore the functionality of human organs, such as a heart valves, in both their healthy and diseased states. This information then provides much-needed insight into the relationships and interactions that occur within the body, providing definitive information in place of what had previously been scientific prediction, thus allowing for better-informed decision-making and management. Furthermore, simulations, such as Fontan surgical planning, can be used as a tool to predict the efficacy of medical procedures and devices used in the treatment of diseases and otherwise malfunctioning organs [1].

Such accomplishments extend beyond the realm of applied surgery, and can be applied to training and education [2], for understanding anatomical changes that lead to disease and how such changes affect healing and functionality [3], even going so far as to predict the level of damage caused by trauma and pinpoint which areas of the brain are possibly affected [4].

This paper provides a review of fluid–structure interaction (FSI) methods using smoothed-particle hydrodynamics (SPH) to analyze the dynamics of biological processes, e.g., heart valve closures, cerebrospinal fluid interacting with brain parenchyma, and so on. The introduction is two-fold. First, we explain why using FSI analyses is preferable, as opposed to single-phase computational fluid dynamics. Second, we succinctly explain the SPH and discuss its suitability for FSI analyses of biomedical applications.

### 1.1. Fluid–Structure Interaction Analyses

The most common numerical method used to perform FSI analyses is the arbitrary Lagrangian–Eulerian (ALE) formulation. The accuracy of ALE methods depends on the use of meshes with high density, which can create issues when modeling gaps between the solid and fluid domains. This is especially difficult when the solid domain is meant to represent a comprehensive patient-specific geometry of high complexity. In models involving detailed anatomy, on top of the complications related to converting medical images into 3D models [5], the use of ALE brings additional complications. For example, ALE methods are either strongly coupled or weakly coupled. Weakly coupled ALE methods are known for accuracy issues when compared to strongly coupled methods and have difficulty performing well when attempting to parallelize the algorithms. Strongly coupled ALE methods yield large ill-conditioned system matrices, i.e., system matrices that are prone to large numerical errors with each system-solving iteration, within each nonlinear iteration, within each time-step. They require the implementation of additional algorithms alongside the ALE algorithm just to keep well-conditioned. As to be expected, increasing numerical operations yields more numerical errors, thus making it nearly impossible to perform effective parallelization of the entire framework [6]. Since the ALE methods rely on the use of meshes with high resolution, the users tend to use simplified geometries to decrease the number of elements necessary for stable calculations. This is done in an attempt to cut processing time down to weeks instead of months, all the while running on supercomputers utilizing thousands of processors while facing convergence issues.

While these mesh-based numerical methods have been used to develop advanced software packages for the analyses of complex problems in both fluid and solid mechanics, they carry their own inherent obstacles, consequently limiting their applicability to other fields [7]. When treating large deformations, which is typical in biomedical applications, it is relatively easier to adopt a discrete set of particles without topological connectivity, i.e., a mesh, to represent the material continuum.

As research in FSI analyses continued to develop, a different approach to modeling, the immersed boundary (IB) method, was introduced by Peskin in 1972 [8]. Peskin delineated the use of the IB method to simulate and calculate patterns of flow through a heart model, which is a solid organ structure. The IB method implements a Lagrangian approach to describe forces, stresses, and deformations of the structure, while utilizing an Eulerian approach for the momentum, viscosity and incompressibility of the fluid–structure system [9]. These equations are then discretized on a Cartesian grid. This is a major advantage of the IB method as grid generation becomes simpler since the body conditions do not need to conform to the grid. Furthermore, since moving boundaries are supported by the Cartesian grid and grid transformations do not require additional terms, the IB method leads to decreased CPU usage, memory, and per-grid-point operation counts [10,11]. These unique properties make the IB method particularly adept at addressing simulations with complex geometries, displacements, deformations, and contact interactions [9,12]. Furthermore, since Peskin’s introduction of IBM, several different approaches have been developed. These include the penalty forcing method, the direct forcing scheme, and the momentum exchange-based IBM [13]. Respectively, the aforementioned methods utilize Hooke’s Law to evaluate the body force on the Lagrangian points [14], resolve the momentum equation at Lagrangian points to update the exerted body forces [15], and allow for the density distribution functions to be interpolated to the boundary points using Lagrangian interpolated polynomials [16]. As IBM continues to progress in the implementation of FSI, Harikrishnan et al. details a novel explicit immersed boundary method that allows for prompt, accurate, and simple interpretation of the no-slip boundary condition [13]. Through the combination of the ALE technique with the lattice Boltzmann Flux solver (LBFS), the solver functions as a potential simulator for moving boundary problems with complex geometry [13]. Looking forward, the development of various solvers for IBM will serve to widen the scope of FSI capabilities.

### 1.2. Biomedical FSI Applications

Many biomedical applications require FSI analyses. Early research by Peskin et al. in 1997 demonstrated FSI interactions in a prosthetic and natural heart valve model with the inclusion of the muscular heart wall. The model demonstrated the ability to apply Navier-Stokes equations to moving solid immersed boundaries [17]. In 2003, Tang et al. used a three-dimensional thick-wall model with FSI to simulate blood flow in the carotid arteries and introduced asymmetric stenosis to quantify the effects of stenosis severity and mimic the pressure conditions on blood flow and artery compression [18]. This technology was then expanded upon, including reconstructed geometries based on CT scans better able to approximate the complicated anatomy of the human artery [19,20]. The use of standard mesh-based numerical methods for biomedical applications remains a challenge and it is still common practice to simplify the computational models by omitting the fluid domain [21]. However, new research has shown the advantage of SPH methods for accurately performing impact simulation, even within the context of blood flow and thrombosis [22]. While mesh-based finite element analyses are classically used in the context of impact biomechanics simulations, the rise of SPH methods in the field is a testament to their status as more than a mere alternative [23].

## 2. Smoothed-Particle Hydrodynamics

Smoothed-particle hydrodynamics is a computational mesh-free Lagrangian method developed by Gingold, Monaghan, and Lucy in 1977, initially intended for use in astrophysics [24,25]. Since then, it has been used for simulating the mechanics of continuum media, such as solid mechanics and fluid flows. It has been used in many fields outside astrophysics, including ballistics, volcanology, and oceanography. In recent years, it has been increasingly adopted by those with an interest in biomedical engineering [26,27,28].

**Figure 1:**
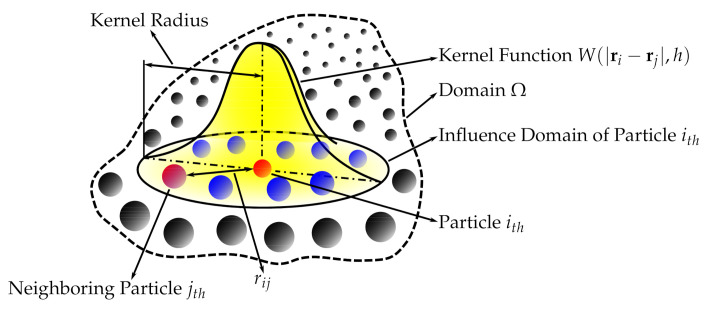
Smoothed-particle hydrodynamics with a kernel approximation.

Succinctly speaking, a continuous field is reconstructed from a cloud of discrete particles. Each of the particles has assigned properties, i.e., mass, pressure, velocity, density (volume). The kernel function is used to encircle several other neighboring particles by the radius of the smoothing length (Figure 1). That means that any property can be reconstructed by taking the volumetric integral of the kernel function multiplied by the local value of a given property, e.g., the pressure $\bar{p}\left(r_{i}\right)=\int p\left(r_{j}\right)W(|r_{i}-r_{j}\left|,h\right){\mathrm{dr}}_{j}$, where $\bar{p}$ is the reconstructed pressure, $r_{i}$ is the position vector, $r_{j}$ is the position vector of the point within the smoothing length radius, *h* is the smoothing length and *W* is the kernel function.

The SPH is governed by a set of ordinary differential equations as follows. The continuity equation, i.e., conservation of mass, is

$$\frac{d\rho_{i}}{dt}=-\rho_{i}\sum_{j=1}^{N_{p}}\left(v_{i}-v_{j}\right)\frac{m_{j}}{\rho_{j}}\cdot \nabla_{i}W_{ij}+4\mathrm{th}\;order\;diffusive\;term,$$

where *W* is the Kernel function and the 4th order diffusive term improves the pressure evolution.

The momentum equation, i.e., Euler equation, is

$$\frac{dv_{i}}{dt}=-\sum_{j=1}^{N_{p}}\left(\frac{P_{i}+P_{j}}{\rho_{i}\rho_{j}}+\Pi_{ij}\right)m_{j}\cdot \nabla_{i}W_{ij}+f_{i},$$

where $\Pi_{ij}$ is the artificial viscosity.

The energy equation, i.e., first law of thermodynamics, is

$$\frac{d\epsilon_{i}}{dt}=-\sum_{j=1}^{N_{p}}\left(\frac{P_{i}}{\rho_{i}^{2}}+\frac{\Pi_{ij}}{2}\right)\left(v_{i}-v_{j}\right)m_{j}\cdot \nabla_{i}W_{ij}.$$

More detail on the numerical methods used in the cardiovascular FSI simulations, e.g., the integration method, artificial viscous term, laminar viscous term, speed of sound used, can be found published elsewhere [29,30].

The SPH interacts with the structural finite elements using a penalty-based contact algorithm. In penalty-based contact, when a penetration is found, a force proportional to the penetration depth is applied to resist and eliminate penetration. Linear contact spring stiffness is based on the nodal masses that come into contact and the time step size as follows

$$k=0.1\frac{m}{\max(\Delta t_{global}^{2},\Delta t_{SPHcontact}^{2})}.$$

The resulting contact stiffness is independent of the material constants so it is well suited for treating contact between fluid and structure. Fluid–structure coupling is achieved by using a position-based Verlet time integration scheme that enforces momentum conservation. Solid particles are assigned a velocity and acceleration which are averaged over a fluid time step to enhance force matching [31].

The combination of the SPH method (used to simulate the fluid domain flow) with a high-order finite element method (used to simulate the solid domain deformations) is ideal for simulating FSI, especially when complex geometries are included. Using SPH methods provides numerical stability because the contact between the solid and fluid domains is easily treated numerically. Moreover, SPH is highly parallelizable. Hence, it is possible to run FSI simulations with geometries complex preserving all their details, and at the same time keeping the simulations numerically stable, accurate, parallelized on a standard GPU workstation (as opposed to large supercomputers), and all of that with a runtime of only hours or days rather than weeks and months.

## 3. Applications

Two notable implementations of SPH-FSI in biomedical applications, namely, blood flow and cerebrospinal fluid flow interactions, have been explored more than other applications. This section is divided accordingly.

### 3.1. Blood Flow in Arteries

The first time SPH was found to be used to replicate a three-dimensional FSI biomechanical process was in 2003, with the introduction of a three-dimensional thick-wall model using FSI to simulate blood flow in the carotid arteries with stenosis [18,32]. Previous mathematical models had been restricted to being one-dimensional and their accuracy was limited because only the average axial velocity and pressure over the cross-section of the tube were calculated [33]. Prior to 2003, higher dimensional models had been used to investigate FSIs in the collapse and ablation of atheromatous plaques in the coronary arteries [34]. They found that wall stress distribution had a very localized pattern and that the dragging force from fluid flow had a considerable effect on wall compression. Other studies formed the foundation for computations for fluid and wall motions in models of arterial stenosis and abdominal aneurysm [35,36]. Stress concentration, found at both edges of the stenosis, was concluded to be responsible for plaque ablation. FSI finite element analysis of pulsatile flow through compliant axisymmetric stenotic arteries was also done and findings showed that severe stenosis causes artery compression, negative flow pressure, and high flow shear stress [37].

More recently, this model has been adopted again, but for simulations of blood flow in patient-specific geometries utilizing CT technology [19,20,38,39]. Furthermore, the particle nature of the SPH method facilitates a convenient platform to model platelets, allowing models to simulate the process of thrombogenesis under the influence of various blood flow parameters [22]. As pointed out throughout this article, the use of SPH is especially justified when the geometries used are complex.

### 3.2. Blood Flow’s Interaction with Heart

In recent years, SPH has been used more commonly to simulate the closure of heart valves. Its purpose is to assess the efficacy of surgical procedures and medical devices. The complexity of heart valve geometries, combined with the large deformations they undergo with every heartbeat between their fully open and fully closed positions, make SPH ideal for conducting computational FSI analyses. The SPH method was demonstrated and validated in several articles on mitral valve closure [30,40,41,42]. Subsequently, it was used to assess several diseased mitral valve states [43,44,45,46] and applications of medical devices designed to correct them [47,48,49,50]. Besides the mitral valve, other valves have been studied using the same methods [42,51,52,53,54,55]. The SPH method has been validated to study the hemodynamics of the left ventricle [29]. Interaction between bioprosthetic heart valves and blood was also studied using SPH [56]. An overview of numerical methods, including SPH, for FSI models of aortic valves, can be found in [57].

### 3.3. Cerebrospinal Fluid’s Interaction with the Brain

The brain, with its intrinsic topology, is the most complex organ in the human body making computational models inherently challenging. The majority of computational models in the literature routinely embed it into lower dimensions. The particle nature of the SPH method allows for a more detailed analysis of the complex neurological structures while having the ability to simulate their interactions with surrounding cerebrospinal fluid.

FSI simulations using SPH are used to demonstrate and to study the cushioning effect of cerebrospinal fluid [58,59,60] and the mechanism of brain injuries induced by an outside loading factor [4,61,62,63]. In the context of disease diagnosis and management, these models are able to assess the risk of developing neurological complications, such as hemorrhage, following treatment in addition to explaining the possible pathophysiology behind the condition itself [64].

Furthermore, the use of SPH is shown to play a role in calculating potential ballistic pathways in forensic investigation. To inform the investigation process, the following are analyzed: the entry wound and blood spatter patterns, the influence of target materials, and the cranial geometry. Using SPH, we can develop a numerical model capable of simulating high-speed ballistic impacts, thus allowing for the standardized evaluation and simulation of “backspatter”, the retrograde ejection of blood and tissue from the entry wound following projectile impact, which can then help determine the proximity of the shooter and potentially differentiate between suicide and homicide [65].

### 3.4. Other Applications

While the SPH method has been more commonly used in heart and brain simulations, other applications have also been explored. As previously stated, the more complex the simulation, the more appropriate it is to use the SPH method. For example, the act of swallowing is a complex process involving soft tissue, muscle, and bone, all of which must be included in order to make the simulations accurate/practical [66,67]. As such, the simulation requires multiple parts whose relationships must be included in the calculation: the soft structures (i.e., pharyngeal wall, soft palate, and tongue) are simulated using a finite element method, bony structures (e.g., mandible, hard palate, and hyoid) are simulated as rigid bodies, and a fluid bolus is simulated using SPH. Mathematical and computational modeling of the stomach is another emerging field of biomechanics where several complex phenomena, such as gastric electrophysiology, fluid mechanics of the digesta, and solid mechanics of the gastric wall, need to be addressed. SPH is a promising approach to model multiphase flows specifically in the gastric lumen [68]. SPH has also been successfully applied in computational modeling of the small intestine [69].

Thus far, all biomedical applications mentioned above are applied to the inside of the human body. However, the interaction of the human body with outside fluid domains can also be considered. For example, an SPH model is used to predict the loading on the human body during elite platform diving [70]. Other studies in human movement science focus on the analysis of stroke technique and the interactions between the water, the paddle, and the kayak [71]. Though technically, that study does not involve direct interaction between the human body and fluid, it does fall under the umbrella of biomedical FSI applications. Furthermore, the body can also interact with, and be damaged by, outside solid objects. Studies looking to understand the anatomical changes that lead to femoral cortical bone remodeling in hip fractures and how such changes affect healing and functionality are one example [3]. As such, the investigation of impact biomechanics is of great interest in the understanding of damages caused by the impact of a projectile with the human body [23].

## 4. Validation

To assess the current state of methods used for simulating fluid flow in an idealized medical device, the U.S. Food and Drug Administration (FDA) has completed a computational fluid dynamics (CFD) inter-laboratory study [72,73], replicated also by our group with special focus on mesh sensitivity analysis [74]. The FDA’s study used a generic medical device consisting of a cylindrical nozzle followed by a sudden contraction and a conical diffuser. Planar particle image velocimetry (PIV) measurements performed at three laboratories were used to validate the data provided by 28 computational results from around the world.

**Figure 2:**
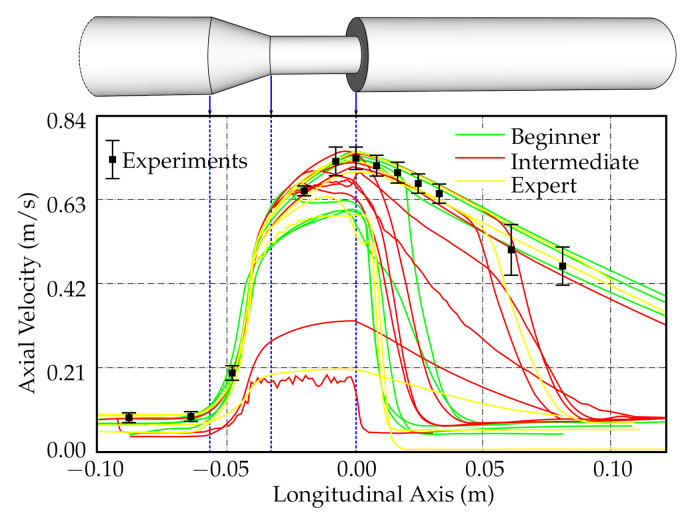
In the Food and Drug Administration (FDA) study [72], the participants were requested to provide simulation data along the model centerline, among others. The results of the study for Reynolds number of 500 are shown here. The graph was re-created based on the data from [75].

To show the results of the above-mentioned study, one of the graphs was re-created based on the data from the “Critical Path” project [75]; see Figure 2. The FDA study-predicted that centerline axial velocities in the entry region and conical contraction were in good agreement with the experimental results, but considerable scatter was observed in the throat region and downstream of the sudden expansion.

Interestingly, a self-ascribed level of expertise by the project participants did not correlate qualitatively with the success of the validation, i.e., comparing axial centerline velocity predicted by CFD to that measured by PIV. Some self-ascribed CFD “experts” produced results with large disagreement when compared to experimental data, while some self-ascribed “beginners” produced results with good agreement when compared to the PIV measurements (Figure 2). This finding demonstrates not only the importance of proper validation but also the necessity of having such validation verified by independent reviews.

A committee on the credible practice of modeling and simulation in healthcare, an interdisciplinary group seeded from a U.S. inter-agency initiative, has recently codified best practices in order to formalize the execution and communication of modeling and simulation practices in healthcare [76]. They suggest ten rules for the credible practice of modeling and simulation in healthcare stated as follows: (1) Define context clearly. (2) Use contextually appropriate data. (3) Evaluate within context. (4) List limitations explicitly. (5) Use version control. (6) Document appropriately. (7) Disseminate broadly. (8) Get independent reviews. (9) Test competing implementations. (10) Conform to standards.

Hence, as demonstrated above, it is crucial to validate numerical results by comparing them with experimental measurements [77,78]. However, there is a multitude of possible measurements that can be chosen for such a comparison. The more complex the geometry, the more the number of variables requiring validation. For example, a mitral valve is composed of two leaflets, namely, the anterior and posterior leaflets. Located in the posterior part of the aortic root, the anterior leaflet has a semicircular shape and is both larger and thicker than the posterior leaflet [79]. It consists of two zones: the rough zone and the clear zone. During systole, the position of the rough zone is adjacent to the posterior leaflet [80,81]. The posterior leaflet, on the other hand, is crescentic with a long circumferential base. Like the anterior leaflet, it can be divided into lateral, central, and medial scallops (referred to as A1, A2, and A3, for the anterior leaflet, and P1, P2, and P3 for the posterior leaflet) [82]. Numerous chordae tendineae, which attach the leaflets to the left ventricle wall via the papillary muscles, connect to each leaflet both along its free edge (primary chordae) and across its ventricular face (secondary chordae).

**Figure 3:**
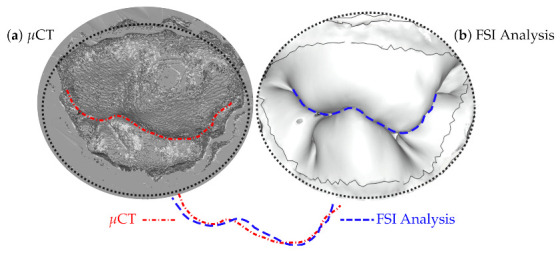
Closed leaflets reconstructed from μCT images (**a**) compared to the results of fluid–structure interaction (FSI) simulations (**b**), after balancing all the structures involved [40]. The curves represent the coaptation line where the posterior and anterior leaflets are in contact.

Competent mitral valve closure during cardiac contraction depends on a well orchestrated force balanced across these structures. Therefore, if the model is validated with respect to one of these structures, it does not necessarily mean that the valve closure is validated, as other parts can still disturb the balance. However, since a balance across these structures yields a closure, validating the closure should validate the model; see Figure 3 [40]. Even in this context, however, the validation is not necessarily guaranteed, due to the theoretical (although unlikely) possibility that different values across multiple structures could still yield the same closure.

## 5. Discussion

Since biomedical applications typically involve multiple deforming solid domains, stochastic processes in addition to FSI, numerical simulations become complex, expensive, and as a result, often inaccurate. Thus, the use of standard mesh-based numerical methods for biomedical applications remains a challenge. Therefore, the use of SPH is being increasingly adopted, especially when the geometry used is of a complex nature. As mentioned several times above, the more complex the application the more the use of SPH is justified (even necessary).

Modeling and simulations can be effectively used to understand and predict the trajectories of physiological processes associated with disease and injury. Their predictive nature is meant to support and inform clinical decisions. However, the information conveyed by said simulations is meant to supplement decision-making, not dictate it, and inappropriate or otherwise ill-placed trust in simulations may result in negative outcomes. Therefore, validation is crucially important in order to ensure that the information conveyed is as accurate as possible.

This review discusses the FSI of biomedical applications. In each of the studies discussed, the solid domain was simulated using a mesh-based finite element method while the fluid domain was simulated using SPH (the mesh-less method). Though not studied in this review, current studies are showing how SPH can also be used to simulate solid domains [83,84]. Thus, FSI simulations where both the fluid and solid domains are simulated using SPH are currently underway.

The ultimate goal is to develop realistic computational models to predict human biological processes. It is often referred to as “computational medicine”. The purpose of computational medicine is to provide means of assessing the function of our organs in both health and disease. There is a multitude of possible diseases that can affect our organs and we need these models to guide us through such inherently complex systems. An example of the application of computational medicine is in the planning and treatment of the neurological syndrome, Moyamoya disease (MMD). Several studies delineate that the traditional treatment approach for treating MMD is through bypass surgery [85]. This intervention prevents ischemic and hemorrhagic stroke in patients by enhancing cerebral blood flow through newly constructed anastomoses, which lower the hemodynamic stress in an abnormal internal carotid system. Most researchers and clinicians focus on identifying improvements in moyamoya vessels on post-bypass angiography, clincal data, and morphometric analysis [85]. However, there is little research and emphasis on the hemodynamic analysis, which has provided improved understanding of stress on the cerebral vasculature. Cerebal FSI have been previously used to identify blood flow patterns and pressure drops in various malformations such as aneurysms [85]. Although these are pioneering advancements, only after we are confident enough in these models, they will be applied in clinical practice more.

As mentioned above, the benefit of using SPH-FSI is that it reduces processing time to only hours or days, rather than weeks and months, while preserving geometry [86]. In a time-sensitive clinical setting, patients’ conditions are everchanging and sometimes require urgent intervention. Though promising, conventional SPH methods are associated with low accuracy within high order approximation schemes. There are several approaches that have been proposed to improve SPH approximation’s accuracy including the finite particle method (FPM), the corrective particle method (CSPM), the kernel gradient free SPH (KGF-SPH), and the decoupled finite particle method (DFPM) [87]. We believe that it is axiomatic that the models, first and foremost, need to be both accurate and realistic. This goal is challenging. Nonetheless, progress is being made across several medical fields [88].

## Data Availability

Data sharing not applicable.

## Abbreviations

The following abbreviations are used in this manuscript:

FSI	Fluid–structure interaction
SPH	Smoothed-particle hydrodynamics
FDA	U.S. Food and Drug Administration
PIV	particle image velocimetry
ALE	Artitrary Lagrangian–Euletian
CT	Computed tomography
IB	Immersed boundary
GPU	Graphics processing unit
CPU	Central processing unit
LBFS	Lattice Boltzman flux solver
CFD	Computational fluid dynamics
MMD	Moyamoya disease
FPM	Finite particle method
CSPM	Corrective particle method
KGF-SPH	Kernel gradient-free SPH
DFPM	Decoupled finite element method
